# Successful Use of Veno-Venous Extracorporeal Membrane Oxygenation in a Patient With Severe COVID-19 Pneumonia

**DOI:** 10.7759/cureus.11938

**Published:** 2020-12-06

**Authors:** Taha Mallick, Mark Barakat, Trevaughn R Baptiste, Mahera Hasan, Ryan Engdahl

**Affiliations:** 1 Surgery, Harlem Hospital Center, New York, USA; 2 Surgery, St. George's University School of Medicine, St. George's, GRD; 3 Surgery, Dow Medical College, Dow University of Health Sciences, Karachi, PAK

**Keywords:** covid-19, ecmo, ards, il-6

## Abstract

Lung damage in coronavirus disease 2019 (COVID-19) pneumonia may be so severe that management with lung-protective ventilation, neuromuscular blockade, and proning cannot sustain life. Extracorporeal membrane oxygenation (ECMO) may allow patients with acute respiratory distress syndrome (ARDS) to undergo a period of lung recovery before being transitioned back to mechanical ventilation. A successful outcome requires both timely initiation of ECMO before development of irreversible organ injury from severe ARDS and selection of patients with adequate physiologic reserve.

We present a 40-year-old healthy male patient with severe COVID-19 pneumonia not responsive to more conservative options for ARDS management. Veno-venous extracorporeal membrane oxygenation (VV-ECMO) rescue therapy was instituted and after 34 days he was successfully decannulated and eventually discharged from the hospital in good condition.

Despite needing ECMO for longer than what is reported in most case reports and series involving patients with COVID-19 pneumonia, our patient made a complete recovery. He was also followed up in an outpatient setting and seen to be doing well.

With appropriate patient selection and timely initiation of ECMO, many patients stand to benefit from this treatment. Ensuring that therapy be delivered to these patients when the need arises requires meticulous planning and provision of the appropriate resources. In addition, inflammatory markers may serve as a further guide to decision-making in patients already on ECMO as has already been indicated in the literature.

## Introduction

As we continue to learn about the spectrum of disease caused by coronavirus disease 2019 (COVID-19), several management strategies have been proposed to improve outcomes. Extracorporeal membrane oxygenation (ECMO) has long had a role in the management of acute respiratory distress syndrome (ARDS) not responsive to more conservative management. With the high prevalence of ARDS in COVID-19 patients, provision of ECMO has become an important concern in several hospitals with the capability of doing so.

We present a healthy patient who developed severe ARDS and did not respond to management with lung-protective ventilation and neuromuscular blockade. He was then started on rescue ECMO therapy with which he made a full recovery to hospital discharge.

## Case presentation

**Figure 1:**
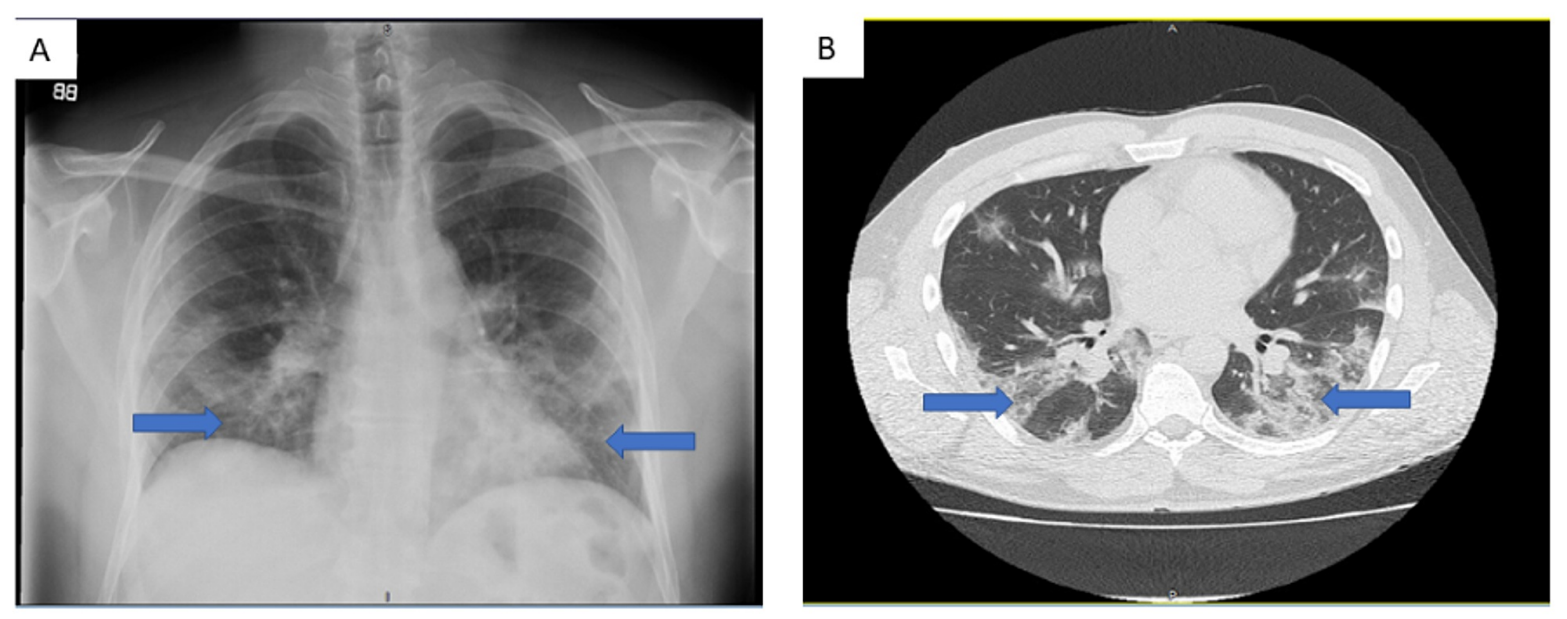
Imaging findings (a) Chest X ray and (b) CT scan findings on admission showing bilateral ground glass pulmonary opacities (blue arrows).

Our patient was a 40-year-old male who presented to the emergency department (hospital 1) with a six-day history of fever, dyspnea on exertion, nausea and vomiting. He denied any history of smoking and had not travelled recently. He had no significant past medical history. On admission, his white blood cell (WBC) count was 7.85x10^3^/uL with absolute lymphocyte count 1.07x10^3^/uL (13.6%) and absolute neutrophil count 6.46 x10^3^/uL (82.3%). C-reactive protein (CRP) level was 146.28 mg/L. Chest X-ray and chest CT imaging revealed bilateral ground glass opacifications concerning for viral pneumonia (Figure 1). He was admitted and tested positive for COVID-19 on reverse transcriptase polymerase chain reaction (PCR) testing of a nasopharyngeal swab specimen. He was started on ceftriaxone, azithromycin and hydroxychloroquine (hospital day 2 to 7). The patient also received oral vitamin C 1000 mg/day (hospital day 4 to 14) and oral zinc sulfate 220 mg (hospital day 5).

**Figure 2:**
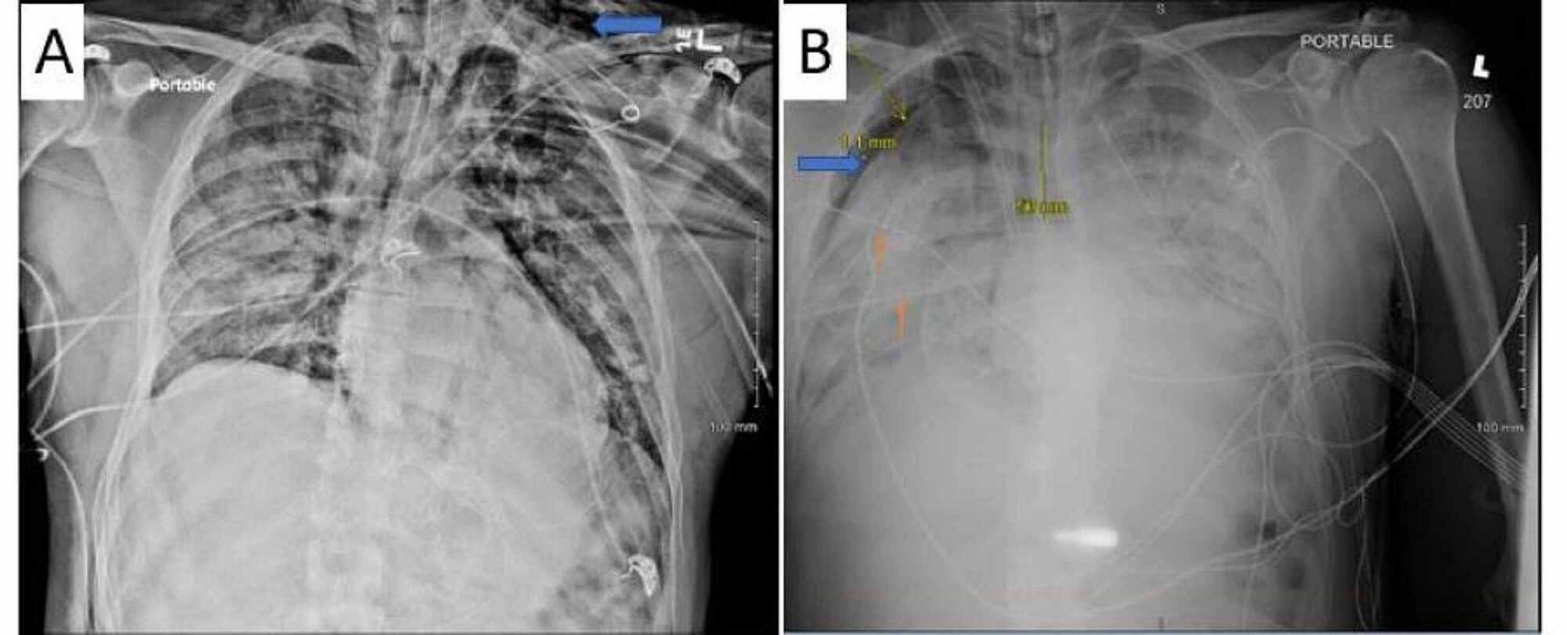
(A) Chest radiograph showing left sided subcutaneous emphysema {blue arrow} (B) Persistent right sided pneumothorax (blue arrow) even with placement of two right sided chest tubes (orange arrows). Also noted on this image is the right internal jugular extracorporeal membrane oxygenation (ECMO) cannula.

He developed worsening respiratory failure and required intubation and mechanical ventilation on admission day 3. Lung-protective ventilation was used with a positive end-expiratory pressure (PEEP) of 5 cm H_2_O and fraction of inspired oxygen (FiO_2_) 100%. His PEEP was increased to 18 cm H_2_O due to low arterial oxygen tension. On hospital day 5, the patient developed subcutaneous emphysema suggestive of left pneumothorax (Figure 2A) and so had a left chest tube placed. Neuromuscular blockade with cisatracurium was initiated on hospital day 6 due to persistent hypoxemia and patient-ventilator dyssynchrony. On hospital day 13, chest X-ray showed a large right pneumothorax but despite placement of two right-sided chest tubes lung re-expansion could not be achieved (Figure 2B). Arterial blood gases showed a partial pressure of oxygen (PaO_2_) < 60 mmHg despite an FiO_2_ of 100% and PEEP of 20 cm H_2_O. His partial pressure of carbon dioxide (PaCO_2_) rose above 70 mmHg with pH maintained higher than 7.30. At this point, the patient was transferred to another facility within our network with ECMO capabilities (hospital 2).

**Figure 3:**
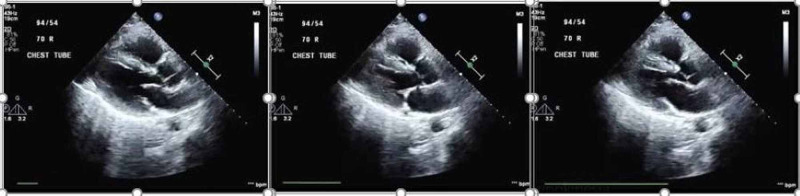
Echocardiogram showing right ventricular free wall hypokinesia

On arrival to hospital 2, he underwent immediate cannulation for femoro-jugular VV-ECMO and was started on an initial flow rate of 4100 ml/min, gas sweep of 11 L/min, goal oxygen saturation (SaO_2_) > 85% and PaO_2_ > 55 mmHg. He was also started on inhaled nitric oxide (iNO) at 20 ppm. Sedation and neuromuscular blockade were continued. A five-day course of dexamethasone (20 mg IV once daily) was given as well. A transthoracic echocardiogram demonstrated mild right ventricular dilation with hypokinesia of the mid-right ventricular free wall and left ventricular ejection fraction of approximately 60% (Figure 3). 

While on ECMO, labs including arterial blood gas (ABG), complete blood count (CBC), chemistries and coagulation parameters were drawn every four to six hours. His PaO_2_ improved over the next few days and his ventilator settings were decreased to facilitate lung recovery using low tidal volumes or pressure support as needed. Anticoagulation was maintained using a heparin drip target anti-Xa level of 0.3-0.7 U/mL, followed by bivalirudin infusion with target partial thromboplastin time (PTT) 50-65 seconds. Red blood cells were transfused as needed to maintain hemoglobin (Hb) above 7 g/dL.

While on ECMO one of his right chest tubes was removed due to dislodgement, following which he developed another right-sided pneumothorax requiring placement of another right-sided chest tube. On hospital day 20, he developed a large right hemothorax and underwent thoracotomy with evacuation of 3 liters of blood from the right chest. Hemostasis was secured and all three chest tubes were replaced. A bronchoscopy with pulmonary toilet and tracheostomy were also performed at this time due to anticipated need for prolonged ventilation.

Despite this, the patient’s clinical course continued to improve with PaO_2_ maintained in target range while ECMO settings were gradually decreased along with weaning down of ventilator FiO_2_. Neuromuscular blockade was successfully discontinued on hospital day 27 (ECMO day 14). Inhaled nitric oxide was stopped on hospital day 28 (ECMO day 15). The patient was removed from the ECMO circuit and decannulated on hospital day 47 (ECMO day 34) and continued on mechanical ventilation. Ventilator settings were successfully weaned, and the patient was ultimately taken off mechanical ventilation and started on oxygen by tracheostomy mask with FiO_2_ 40% on hospital day 59 (totaling 57 days on mechanical ventilation) followed by serial removal of bilateral chest tubes. His tracheostomy was successfully decannulated on hospital day 69 and he was discharged to a rehabilitation facility on hospital day 75. He was followed up in an outpatient setting three weeks after discharge and continued to do well. 

CRP levels varied between 58.87 mg/L - 367.4 mg/L (highest level noted on hospital day 35, ECMO day 23). White blood cell and neutrophil count reached a maximum of 52.33 x 103/uL and 30.59 x 103/uL respectively on hospital day 44 (ECMO day 31) which was at the time he developed a hemothorax. Besides this peak, the highest white blood cell and neutrophil counts were 34.99 x 103/uL and 22.37 x 103/uL respectively on hospital day 19 (ECMO day 7) and dropped to 11.2 x 103/uL and 10.05 x 103/uL on discharge. His lowest lymphocyte count was 0.43 x 103/uL on hospital day 36 (ECMO day 24). Interleukin 6 (IL-6) level was noted to be 64.5 pg/ml (hospital day 15, ECMO day 2).

A timeline of significant events can be seen below in Table 1.

**Table 1:** Timeline of significant events ECMO: extracorporeal membrane oxygenation

Date	Hospital day	ECMO day	Event
3/27/2020	1	N/A	Admitted to Hospital 1
3/29/2020	3	N/A	Endotracheal intubation
3/31/2020	5	N/A	Insertion of left chest tube
4/1/2020	6	N/A	Initiation of neuromuscular blockade
4/1/2020	13	N/A	Insertion of 2 right chest tubes
4/1/2020	14	1	Initiation of ECMO at Hospital 2 Initiation of inhaled nitric oxide
4/1/2020	20	6	Thoracotomy and tracheostomy in operating room
4/1/2020	27	15	Discontinuation of neuromuscular blockade
4/1/2020	28	16	Discontinuation of inhaled nitric oxide
5/12/2020	47	35	Decannulation from ECMO
5/24/2020	59	N/A	Discontinuation of mechanical ventilation
6/3/2020	69	N/A	Decannulation of tracheostomy
6/9/2020	75	N/A	Discharged to rehabilitation facility

## Discussion

As the COVID-19 pandemic continues to affects various parts of the globe, a lot of literature has emerged on various aspects of the disease process. Two epidemiologic studies reviewed by us indicate the median age of infected patients to be 49 and 56 years, respectively, with men comprising 73% and 54% of patients, respectively [1,2]. Studies indicate that involvement of the lower respiratory tract in the form of bilateral pneumonia or pulmonary infiltrates on imaging is seen in 75-100% of patients [1,2]. Up to 29% of patients with COVID-19 may develop ARDS [1]. Patients with COVID-19 requiring intensive care unit (ICU) admission have a mortality as high as 61.5% at 28 days [3].

Management of ARDS in COVID-19 is similar to that of ARDS arising from other etiologies [4]. A trial of high-flow nasal oxygen may be considered along with close monitoring for deterioration. In mechanically ventilated patients management includes lung-protective ventilation, proning, sedation, neuromuscular blockade and in some cases iNO. In patients who remain unable to achieve adequate oxygenation or carbon dioxide removal ECMO allows time for lung recovery while providing oxygenation of blood and removal of carbon dioxide and so may allow us to salvage these patients. Current criteria for initiation of VV-ECMO in ARDS patients are derived from the EOLIA trial [5] which are as follows: PaO_2_:FiO_2_ ratio < 50 mmHg for > three hours or PaO_2_:FiO_2_ ratio < 80 mmHg for > six hours or arterial pH < 7.25 with PaCO_2_ > 60 mmHg for > six hours. Current guidelines from the Extracorporeal Life Support Organization (ELSO) [6] recommend use of ECMO in patients who have failed more conservative management as outlined above and meet the criteria outlined in the EOLIA trial. There is no definition of futility of treatment but no cardiac or lung recovery with 21 days on ECMO may be used as an indication to stop ECMO with return to mechanical ventilation [6]. However exceptions must be made based on circumstances.

Several case reports and case series have been published indicating the potential for use of ECMO in COVID-19 patients. A case series from nine US-based hospitals comprising 32 patients with COVID-19 on ECMO showed that 22 out of 32 patients were alive with five having been decannulated. Of those who survived decannulation, all received VV-ECMO with mean and median of 8.4 and eight days on ECMO respectively. Four out of five of those that survived decannulation received IV steroids. Ten out of 32 patients died before or shortly after decannulation including one patient who died from intracranial hemorrhage while on ECMO. There were no significant differences between the five patients who survived decannulation and those who died in terms of age, gender, comorbidities or treatment with anti-viral medication (remdesivir), anti-IL-6 monoclonal antibodies or hydroxychloroquine [7]. However, with a larger sample size significant differences may still exist. A recent series from Massachusetts General Hospital presented six patients with COVID-19 who were put on ECMO. One patient died due to a hemorrhagic stroke while five out of the six patients are still alive (survival rate of 83.3%) with four patients having been decannulated and two having been extubated [8].

A separate case series from Shanghai, China [9] found that of eight patients who received ECMO, four died (including one who received three hours of extracorporeal cardiopulmonary resuscitation (ECPR)-veno-arterial (VA) ECMO) while four are alive of which three have been decannulated but not weaned off mechanical ventilation while one remains on VV-ECMO. Excluding the patient requiring ECPR, patients received four to 21 days of mechanical ventilation before ECMO and 18-47 days of ECMO. Two further case series of COVID-19 patients from China show poorer outcomes with death reported in five out of six and three out of three patients requiring ECMO respectively [3,10].

The substantial variability of outcomes with ECMO between different studies may indicate the need for further refinement of criteria to improve patient selection as well as allowing for early initiation of ECMO in these patients to maximize their chances of recovery. In addition, a careful evaluation of contraindications to initiation of ECMO may help select patients most likely to benefit from this resource-intensive form of therapy.

Several studies have shown statistically significant differences in lab parameters between COVID-19 survivors and non-survivors [11] and a correlation of these parameters with severity of infection [12]. This includes higher levels of several inflammatory markers such as CRP, IL-6, neutrophil count/percentage and lactic acid dehydrogenase (LDH) and lower lymphocyte counts. In addition, in patients on ECMO (for COVID-19 or other causes) both IL-6 and CRP levels have been shown to be higher among non-survivors lending them prognostic significance [13]. Accordingly, there has been interest in the use of anti-inflammatory agents including anti-IL-6 monoclonal antibodies. One case report in the literature in fact studies changes in inflammatory mediators, ventilator settings and P/F ratio with administration of tocilizumab and vitamin C with temporal improvements in these parameters demonstrated graphically. This particular patient required 160 hours of VV-ECMO [14]. Similarly lymphopenia may indicate a poorer prognosis in patients with COVID-19 requiring ECMO as this therapy is known to cause a further drop in lymphocyte count [15]. The extent to which various anti-inflammatory therapies, anti-viral medications, and other treatment adjuncts are effective in severe COVID-19 pneumonia remains to be answered.

Experience also exists from previous outbreaks of respiratory tract viruses such as the swine flu (H1N1) and Middle East respiratory syndrome (MERS) outbreak [16]. A review of the literature indicates that criteria for initiation of ECMO were similar to that currently advocated for COVID-19 patients. In the case of H1N1, survival rate on ECMO were reported to be 35.7 - 86.8% while in MERS it was noted to be 31.6 - 61.5% in different case series. In both outbreaks no definite survival benefit was noted with ECMO but systematic reviews indicate that in younger healthy patients there appears to be a benefit with timely initiation of ECMO.

VA-ECMO and ECPR are also modalities that may be considered. Results with VA-ECMO in the literature appear to be poorer probably because patients are sicker with cardiogenic shock in addition to respiratory failure. In the two case series reviewed by us [7,9] no patients with COVID-19 requiring VA-ECMO or ECPR survived.

Like any other form of treatment ECMO does have complications, including those arising from the cannulation process, infections, hemorrhagic and thrombotic complications [17]. Intracranial hemorrhage [18] is a particularly feared complication and may arise from the need to anti-coagulate patients on ECMO. In the EOLIA trial, ECMO was associated with an increased need for transfusions due to bleeding and increased risk of thrombocytopenia. ECMO may also give rise to an inflammatory response [19]. 

Our case is that of an otherwise healthy patient who developed severe ARDS and bilateral pneumothoraces with inability to maintain oxygenation with lung-protective ventilation and neuromuscular blockade. It must be noted that proning was not implemented in our patient which is an considered an essential step in the management of severe ARDS. Instead, due to his rapid deterioration, transfer to a suitable facility with initiation of rescue ECMO was prioritized. Our patient did not at any point require dialysis and had good cardiac function at the time of initiation of ECMO. He therefore seemed to be an appropriate candidate for ECMO. In addition, it must be noted that he responded well to initiation of ECMO being able to maintain PaO_2_ in target range while ventilator settings were decreased which probably helped facilitate lung recovery. His CRP, WBC and neutrophil levels showed an appropriate decline while his lymphocyte count improved while on ECMO indicating that he was progressing towards a favorable outcome. IL-6 levels were not trended in this patient. All these factors helped justify allowing this patient to remain on ECMO for 34 days with an ultimately successful outcome. Finally, while our patient did receive a five-day course of steroids it must be noted that he did not receive other adjunctive therapies such as anti-IL-6 monoclonal antibody treatment or remdesivir. However, these treatments may still play a role in COVID-19 patients on ECMO and further research will be needed to elucidate this.

## Conclusions

ECMO remains a valuable resource in the management of severe COVID-19 pneumonia not responsive to more conservative options for ARDS management. By allowing for a period of lung recovery, this modality may provide patients who would otherwise succumb to the disease process a chance to make a good recovery. However timely initiation of ECMO therapy and appropriate patient selection remains paramount to successful outcomes. Trending of inflammatory markers during ECMO may also help guide decision making for these patients.
